# Rac1 and Rac3 GTPases Regulate the Development of Hilar Mossy Cells by Affecting the Migration of Their Precursors to the Hilus

**DOI:** 10.1371/journal.pone.0024819

**Published:** 2011-09-20

**Authors:** Roberta Pennucci, Stefania Tavano, Diletta Tonoli, Sara Gualdoni, Ivan de Curtis

**Affiliations:** Cell Adhesion Unit, Division of Neuroscience, San Raffaele Scientific Institute and San Raffaele University, Milano, Italy; University of Turin, Italy

## Abstract

We have previously shown that double deletion of the genes for Rac1 and Rac3 GTPases during neuronal development affects late developmental events that perturb the circuitry of the hippocampus, with ensuing epileptic phenotype. These effects include a defect in mossy cells, the major class of excitatory neurons of the hilus. Here, we have addressed the mechanisms that affect the loss of hilar mossy cells in the dorsal hippocampus of mice depleted of the two Rac GTPases. Quantification showed that the loss of mossy cells was evident already at postnatal day 8, soon after these cells become identifiable by a specific marker in the dorsal hilus. Comparative analysis of the hilar region from control and double mutant mice revealed that synaptogenesis was affected in the double mutants, with strongly reduced presynaptic input from dentate granule cells. We found that apoptosis was equally low in the hippocampus of both control and double knockout mice. Labelling with bromodeoxyuridine at embryonic day 12.5 showed no evident difference in the proliferation of neuronal precursors in the hippocampal primordium, while differences in the number of bromodeoxyuridine-labelled cells in the developing hilus revealed a defect in the migration of immature, developing mossy cells in the brain of double knockout mice. Overall, our data show that Rac1 and Rac3 GTPases participate in the normal development of hilar mossy cells, and indicate that they are involved in the regulation of the migration of the mossy cell precursor by preventing their arrival to the dorsal hilus.

## Introduction

Rac GTPases regulate several cellular processes including actin dynamics and adhesion [1], and are critical for neuronal development and synaptogenesis [2]–[4]. Two members of the Rac family are co-expressed during development in several neuronal types: the ubiquitous Rac1 and the neural-specific Rac3/Rac1B [5]–[7]. Rac1 and Rac3 GTPases share about 90% protein identity, and their pattern of expression during development differs substantially, suggesting specific functions. Rac1 has been implicated in the regulation of axons, dendrites, and spines [8]–[11]. Recently, conditional deletion of Rac1 in ventricular zone progenitors has indicated a role of Rac1 in axon guidance while axonal outgrowth is not affected [12]. Rac1 is detected from very early embryonic development in the mouse (E7.5), where it is essential from early development, and its KO results in embryonic lethality, with Rac1-null mice dying during early development due to migratory defects [13]. The transcript for Rac3 is already detectable in the mouse nervous system at E13, and is developmentally regulated in the brain, with a peak at time of intense neurite branching and synaptogenesis [14]. While most of the work in primary neurons has been on Rac1, we have recently shown that the double deletion of the two GTPases in developing neurons leads to a strong neurological phenotype when compared to mice with single Rac1 or Rac3 deletion, showing that both GTPases are important for the development of a functional nervous system [15]. Our previous comparative analysis of the phenotype of single and double knockout mice for the Rac1 and Rac3 GTPases has shown a specific defect in the size of the dorsal hilus of the hippocampus that is strongly reduced compared to single knockout or wildtype animals [15]. This reduction correlated with a strong decrease of the hilar mossy cells, an important class of excitatory neurons targeting dentate granule cells [16]. This defect was clearly detectable at P13 (postnatal day 13), when the double mutant mice show evident epilepsy. In contrast, no major morphological defects were evident in the pyramidal neurons of the CA1–CA3 region of the hippocampus, nor in the dentate granule cells of the double knockout mice.

Of the three classes of major excitatory hippocampal neurons, CA1–CA3 pyramidal neurons are born in the ventricular zone at E10.5 [17] with a peak at E13.5, as revealed by double labelling for GluR2/3 (glutamate receptor subunits 2 and 3) and BrdU (bromodeoxyuridine) [18]. Granule cells form over an extensive period during development and in the adult [19]–[21]. The first granule cells are born at E12.5 in the primary dentate neuroepithelium [18], [22]. Despite the important role of hilar mossy cells in the organization of the hippocampal circuitry [16], [23]–[24], limited information is available on their development, due to the lack of markers for the developing immature mossy cells. Calretinin and GluR2/3 staining has been used to label mature mossy cells from P7–P8 [15], [18]. Mossy cells are excitatory glutamatergic neurons that represent the principal cell type in the dentate hilus [25]–[27]. They are involved in a range of physiological and pathological conditions [28]–[29]. Mossy cells are part of the circuitry that plays important regulatory functions in the hippocampus, where they are believed to modulate the signals arriving from the cortex to the dentate gyrus [28]. In this respect, it is known that mossy cells receive excitatory inputs from the dentate granule cell axons, the mossy fibers. These axons project to the pyramidal cells of the CA3, but also send extensive collaterals to the hilar mossy cells. The axons of mossy cells project back to granule cells, thus regulating the flux of information travelling from the cortex through the hippocampus and back to the cortex [23], [26], [27], [30].

Extensive loss of hilar mossy cells has been reported in patients with temporal lobe epilepsy [31]. These neurons represent one of the first neuronal populations to die also in experimental models of epilepsy [29], [32]–[35]. Anatomic studies have suggested that mossy cells originate from the germinative zone immediately adjacent to the primary dentate neuroepithelium where progenitors of dentate granule neurons are born [22], [36]. It has been recently shown that the first wave of granule cell production coincides with the peak of mossy cell generation [18]. The majority of mossy cells are born between E12.5 and E13.5, and their production is severely affected in mutant animals in which granule cell production is disrupted during precursor proliferation or neuronal differentiation, respectively [18]. Based on these findings, these authors have hypothesized that mossy cells and granules may share the same precursors and developmental influences.

In this study we have investigated the mechanisms underlying the loss of hilar mossy cells in mice deficient of both Rac1 and Rac3. The results indicate a defect in the migration of the precursors from their site of origin, and suggest a possible defect in later development of the residual neurons in the hilus, thus indicating the implication of both GTPases in the regulation of distinct aspects of mossy cell development.

## Results and Discussion

### Rac depletion affects the number of mossy cells and proper synaptogenesis in the dorsal hilus

We have recently shown that the combination of the full knockout of Rac3 (Rac3^KO^) with the conditional deletion of Rac1 by a SynI-Cre (Rac1^N^) results in mice with specific double deletion of these GTPases (Rac1^N^/Rac3^KO^) in several developing neurons [15]. The Rac1^N^/Rac3^KO^ mice show specific neurological and anatomical defects when compared to the single mutants. These mice become heavily epileptic and die around the end of the second postnatal week. In contrast to wildtype or single knockout mice (Rac3^KO^ or Rac1^N^), the double knockout mice show a strong decrease of the size of the dorsal hippocampal hilus, with specific decrease of mossy cells at P13 [15]. No evident decrease in GluR2/3-positive mossy cells was detected in the dorsal hilus of P13 single knockout mice for either neuronal Rac1 or Rac3. We have now investigated the possible causes of the lack of mossy cells from the dorsal hilus of mice depleted of both neuronal Rac1 and Rac3.

We have first quantified the number of mossy cells detectable in the dorsal hilus of double mutant mice. Rac3 mRNA is expressed in hilar cells at P7 [6], and remained expressed in most developing hilar neurons at P13 (**Fig. S1, A**). Moreover, histological analysis of SynI-Cre/ROSA26 obtained by crossing the Rosa26 reporter mice with transgenic SynI-Cre mice has shown that at E12.5 the SynI-Cre activity is mostly found in differentiated neurons including hippocampal neurons, while it is not detected in the ventricular regions of the brain [37]. This finding is consistent with the data on the expression of the endogenous Synapsin I [38]. By using the same reporter system, we found that most if not all hilar neurons expressed the SynI-Cre transgene (**Fig. S1, B**). Together, the *in situ* and histological data suggest that hilar mossy cells co-express Rac1 and Rac3. It is therefore expected that the deletion of both genes affects the development of these neurons, while deletion of either gene may be compensated by the activity of the other.

**Figure 1:**
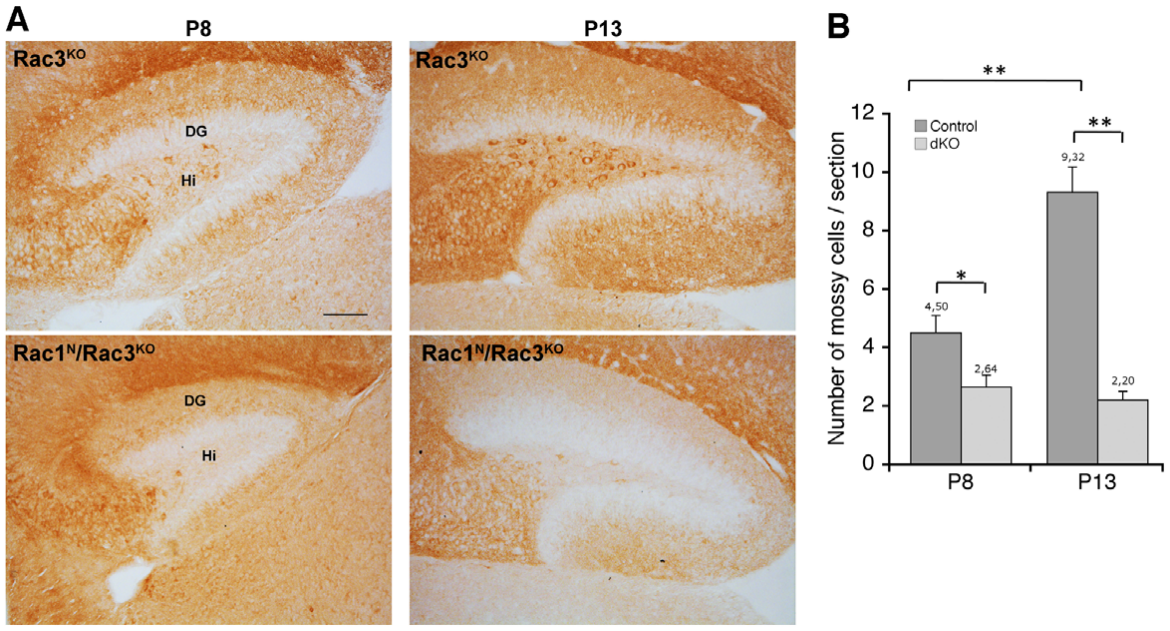
The number of mossy cells is reduced in the dorsal hilus of Rac1^N^/Rac3^KO^ mice. (**A**) Representative immunohistochemistry with anti-GluR2/3 antibody on sagittal sections of the dorsal hippocampus of P8 and P13 control Rac3^KO^ (top) and Rac1^N^/Rac3^KO^ double knockout (bottom) mice. In the hilar region (Hi) staining for GluR2/3 is specific for the mossy cells. DG, dentate gyrus. The number of GluR2/3-positive cells is reduced in Rac1^N^/Rac3^KO^ mice compared to Rac3^KO^ animals at both developmental stages analysed. Scale bar, 100 µm. (**B**) Quantification of GluR2/3-positive cells in the hilus of Rac3^KO^ (Control) and Rac1^N^/Rac3^KO^ mutants (dKO) at P8 and P13. GluR2/3-positive mossy cells were counted in 19–25 sections from 3 mice per genotype per age. Bars are average numbers per section ± SEM. *P<0.05; **P<0.001.

Mouse mossy cells in the dorsal hilus can be recognized by the specific expression of GluR2/3 that starts to be clearly detectable in P7/P8 mice [15], [39]. It is to be noted that this is so far the main reliable specific marker for the mouse mossy cells within the dorsal hilus, since another established marker of mossy cells, the calcium-binding protein calretinin is only expressed in the cell body of ventral mossy cells [39], while it is mainly limited to the axon of dorsal mossy cells [15], [40], [41]. We found a 40% decrease in the number of mossy cells in P8 double knockout mice compared to Rac3^KO^ littermates. The reduction became 75% in the hilus of P13 Rac1^N^/Rac3^KO^ mice (**Fig. 1**). Interestingly, the number of GluR2/3-positive cells was not significantly changed between P8 and P13 in the double knockout mice, while the number of cells expressing GluR2/3 doubled in the control animals.

**Figure 2:**
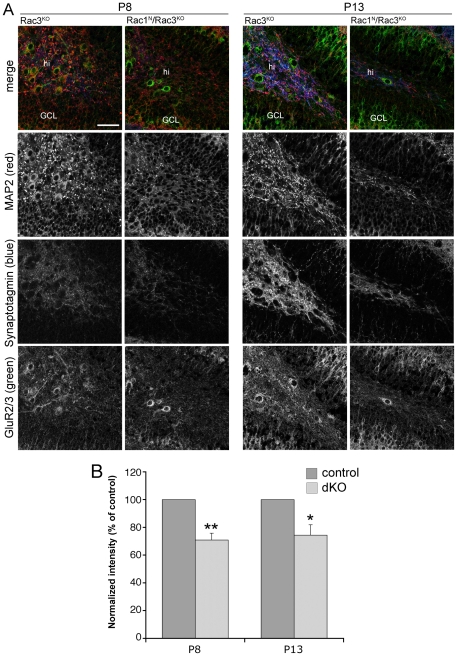
Synaptogenesis is affected in the hilus of double knockout mice. P8 and P13 sagittal sections of dorsal hippocampi were immunostained for the indicated antigens. (**A**) Confocal images of MAP2 (red), GluR2/3 (green), and synaptotagmin 1 (blue) immunoreactivity within the hilus of control Rac3^KO^ and Rac1^N^/Rac3^KO^ double knockout littermates. The three markers are expressed in the hilus of P8 and P13 control animals, and they are strongly reduced in the hilus of P8 and P13 double KO mice, respectively. GCL, granule cell layer of the dentate gyrus; hi, hilus. Bar: 50 µm. (**B**) Quantification of synaptotagmin 1 fluorescence intensity in the hilar neuropil of Rac3^KO^ (control) and Rac1^N^/Rac3^KO^ (dKO) littermates at P8 and P13, respectively. The signal for synaptotagmin 1 was reduced in the double knockout mice, both at P8 and P13. Bars are average values from 13 (P8) or 10 (P13) sections from 3 mice per genotype. *P<0.05; **P<0.001.

We have next analyzed the possible effects of Rac depletion on the development of the mossy cells that were able to reach the hilus in the double knockout mice. We have previously shown that Rac1^N^/Rac3^KO^ mice have a strong impairment of synaptogenesis and dendritogenesis at P13 [15]. Here we have investigated whether these defects were already present at P8, when differentiating GluR2/3-positive mossy cells are first clearly detectable. The reduction in mossy cells was paralleled by the reduction of the signal for the somato-dendritic marker MAP2 [42] and for the presynaptic marker synaptotagmin 1 [43] in the hilus of P8 and P13 double knockout mice (**Fig. 2, A**). In particular, quantification of the fluorescence intensity for synaptotagmin 1 showed a reduction of approximately 30% both at P8 and P13 (**Fig. 2, B**). Higher magnification confocal images showed that both dendritic arborization and presynaptic inputs around mossy cells were strongly decreased either at P8 or P13 (**Fig. S2**). In particular, the axonal terminals of the dentate granule cells identifiable by ZnT-3, a zinc transporter specific of the mossy fibers [44], [45], were already evident in the dorsal hilus of P8 control mice, but strongly reduced in Rac1^N^/Rac3^KO^ littermates (**Fig. S3**). We have previously shown that the signal for ZnT-3 was strongly reduced in the dorsal hilus of P13 double KO mice, while targeting of mossy fibers to the stratum lucidum of hippocampal CA3 was not evidently affected [15]. Since ZnT-3-positive collaterals of the granule cell mossy fibers represent a major input to mossy cells, these results show that the developing circuitry of the hippocampus is already affected in P8 double knockout animals. Moreover, although these data do not directly show a role of Rac1 and Rac3 in the late development of mossy cells, they suggest that the two GTPases may be implicated in the differentiation of the precursors after their arrival to the hilus.

### Cell death is not significantly altered in the dorsal hippocampus of Rac1^N^/Rac3^KO^ mice

**Figure 3:**
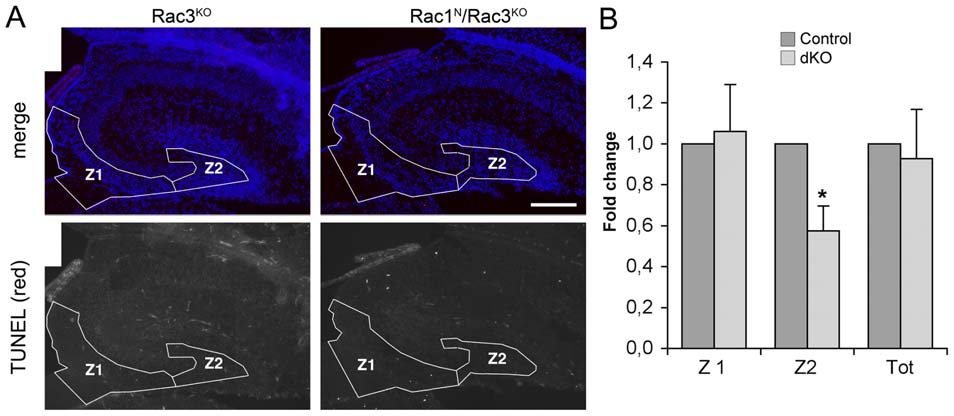
Double knockout of Rac1 and Rac3 does not enhance cell death. (**A**) Sagittal brain sections including the dorsal hilus from P0 Rac1^N^/Rac3^KO^ and Rac3^KO^ control mice were treated for fluorescent TUNEL staining (red), and DAPI (blue). TUNEL-positive cells were counted in zone 1 (Z1) including the VZ/SVZ along the lateral ventricular wall and the DMS, and zone 2 (Z2) including the hilar region. Scale bar: 200 µm. (**B**) Quantification of cell death in mice from different postnatal stages: bars are normalized means ± SEM of the number of TUNEL-positive cells per section (n = 5 different stages considered: P0, P4–P7). Analysis was performed on 30 sections from a total of 8 mice per genotype.

Although a role of the Rac GTPases in the differentiation of hilar mossy cells can not be ruled out, our results indicate that additional mechanisms may cause the reduction in the number of mossy cells from the dorsal hilus of the double knockout mice. We have tested the hypothesis that these neurons die before or after reaching the hippocampus. Developing mossy cells are believed to migrate from their site of origin in the VZ/SVZ (ventricular/subventricular zone) of the hippocampal primordium, through the DMS (dentate migratory stream), to the hilus [18]. Cell death was therefore assessed by TUNEL staining in the VZ/SVZ/DMS (defined as zone 1), and in the hilus (defined as zone 2). Very few TUNEL-positive cells were observed in these areas at any of the stages analyzed in either control or Rac1^N^/Rac3^KO^ mice (**Fig. 3, A** **; Fig. S4**). Given the very small numbers of TUNEL-positive cells per section found in both control and double knockout hippocampi, we pooled the analysis performed at different stages between P0 and P7, when the reduction of the hilus was first detected [15]. Analysis of 30 sections from a total of 8 mice per genotype were analyzed for cell death by TUNEL staining. Quantification of TUNEL-positive cells showed that cell death was not increased in the VZ/SVZ/DMS and hilar regions of double knockout mice. On the contrary, a decrease in cell death was observed in the hilar region of double knockout mice (**Fig. 3, B**). It is to mention though that this reflected a change on a small number of cells detected in the samples analyzed (an average of 1,4 TUNEL-positive cells per section from control brains versus 0,8 TUNEL-positive cells per section from brains from Rac1^N^/Rac3^KO^ mice). Overall these data indicate that cell death does not account for the strong decrease in mossy cell number observed in the dorsal hilus of Rac1^N^/Rac3^KO^ mice.

### Decreased numbers of BrdU-positive mossy cell precursors are detected in the hilus of P8 and P13 double knockout mice

**Figure 4:**
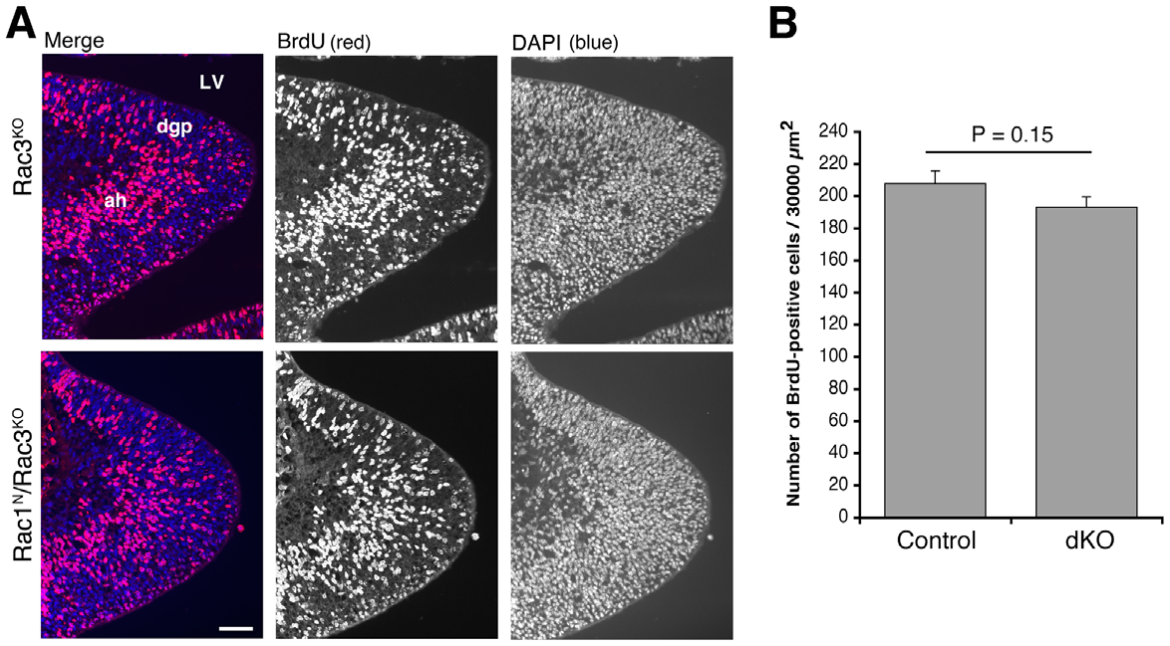
Precursor proliferation is not altered in the hippocampal primordium of E12.5 Rac1^N^/Rac3^KO^ mice. (**A**) Immunostaining for BrdU (red) on sagittal sections of the hippocampal primordium from E12.5 Rac3^KO^ (top) and Rac1^N^/Rac3^KO^ (bottom) embryos. Blue, nuclear staining by DAPI. Scale bar, 50 µm. Abbreviations: ah, ammonic neuroepithelium; dgp, primary dentate neuroepithelium; LV, lateral ventricle. (**B**) Quantification of BrdU-labeled cells in the hippocampal primordium of E12.5 Rac3^KO^ (Control) and Rac1^N^/Rac3^KO^ (dKO) littermates. BrdU-positive cells were counted in the area of the hippocampal primordium including the dgp. Bars are means ± SEM (n = 12–13 sections from 2 mice per genotype).

The lack of a detectable increase in cell death suggests that the severe decrease in the number of mossy cells observed in the dorsal hilus of double knockout mice may be a consequence of the fact that mossy cell precursors can not reach their final destination in the hilus. It has been previously shown that the production of mossy cells in the hippocampal primordium peaks around E12.5, and coincides with the first wave of granule cell production [18]. We first examined whether neuronal precursors proliferate properly in Rac1^N^/Rac3^KO^ mice. Pregnant female mice of gestational age E12.5 were injected with BrdU and sacrificed 2 hours after injection. Quantification of the density of BrdU-positive cells in the hippocampal primordium (**Fig. 4, A**) showed that precursor proliferation was not altered in the double knockout mice compared to control littermates (**Fig. 4, B**). Therefore, the depletion of Rac1 and Rac3 did not evidently affect the number of BrdU-labelled cells in the hippocampal primordium at E12.5, suggesting that gross alteration in precursor proliferation was not altered at this stage.

The negative results obtained from the analysis of cell death and proliferation exclude these events as major causes of the hilar phenotype observed in the Rac1^N^/Rac3^KO^ mice. Given the high number of BrdU-positive cells within the hippocampal primordium, which includes precursors for both granule cells and less numerous mossy cells, we can not exclude that possible differences in the number of mossy cell precursors between double and single knockout mice remain undetectable under the experimental conditions examined. On the other hand, the previous finding that at E12.5 the SynI-Cre activity is detected in differentiated neurons but not in the ventricular regions of the brain [37] suggests that Rac1 is not deleted yet at the stage when mossy cells are born.

**Figure 5:**
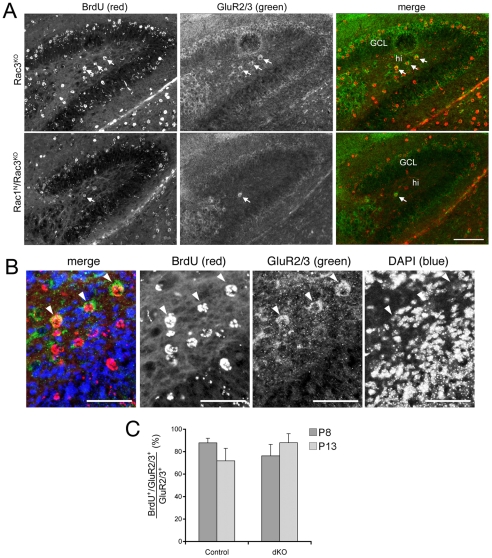
Most GluR2/3-positive mossy cells derive from E12.5–E13 BrdU-labelled precursors. (**A**) Sagittal sections of the dorsal hippocampus from P8 mice labelled with BrdU at E12.5–E13. Immunostaining shows sections from Rac3KO (top) and Rac1^N^/Rac3^KO^ (bottom) mice stained with antibodies for BrdU (red) and GluR2/3 (green). Arrows: BrdU-positive hilar mossy cells co-stained for both antigens. GCL, granule cell layer; hi, hilus. Scale bar: 50 µm. (**B**) Three-fold enlargement of the hilar region of the upper panel (Rac3^KO^). Arrowheads show GluR2/3-positive, BrdU-labelled hilar mossy cells. Scale bar: 25 µm. (**C**) Quantification of the percentage of GluR2/3-positive cells that were also BrdU-positive in control and double knockout (dKO) hippocampi from P8 and P13 mice. Bars are means ± SEM (n = 3–5).

We then tested the hypothesis that double Rac depletion affected the migration of mossy cell precursors from the hippocampal primordium to the hilus. For this, we have analyzed the BrdU-positive cells found in the postnatal hilus after labelling by injection of pregnant mice carrying E12.5/E13 embryos [18]. We found that several BrdU-positive cells labelled at E12.5-E13 were detectable in the hippocampal region of P8 mice (**Fig. 5, A**). In particular, we found that more than 80% of the GluR2/3-positive mossy cells were BrdU-positive in the hilus of P8 control mice (**Fig. 5, C**). These data are consistent with previous findings reporting that the majority of mossy cells derive from precursors born around E12.5 [18]. Triple labelling showed that the GluR2/3-positive, BrdU-positive mossy cells were characterized by having a large round nucleus (**Fig. 5, B**), as previously described [40]. We also observed several hilar BrdU-positive cells with large round nuclei that were negative for GluR2/3. These cells may include undifferentiated mossy cell precursors.

The majority of the GluR2/3-positive mossy cells were BrdU-positive also at P13 (**Fig. 5,C**). On the other hand, quantification showed that the percentage of BrdU-positive hilar cells with a large round nucleus with weak DAPI staining that were also positive for GluR2/3 raised from 33% (n = 4; SEM = 7.9%) to 52% (n = 3; SEM = 4.4%) between P8 and P13 control mice, respectively. This relative increase could be explained by the maturation of the precursors between P8 and P13, as indicated also by the increase in the total number of GluR2/3-positive cells between P8 and P13 (**Fig. 1**).

**Figure 6:**
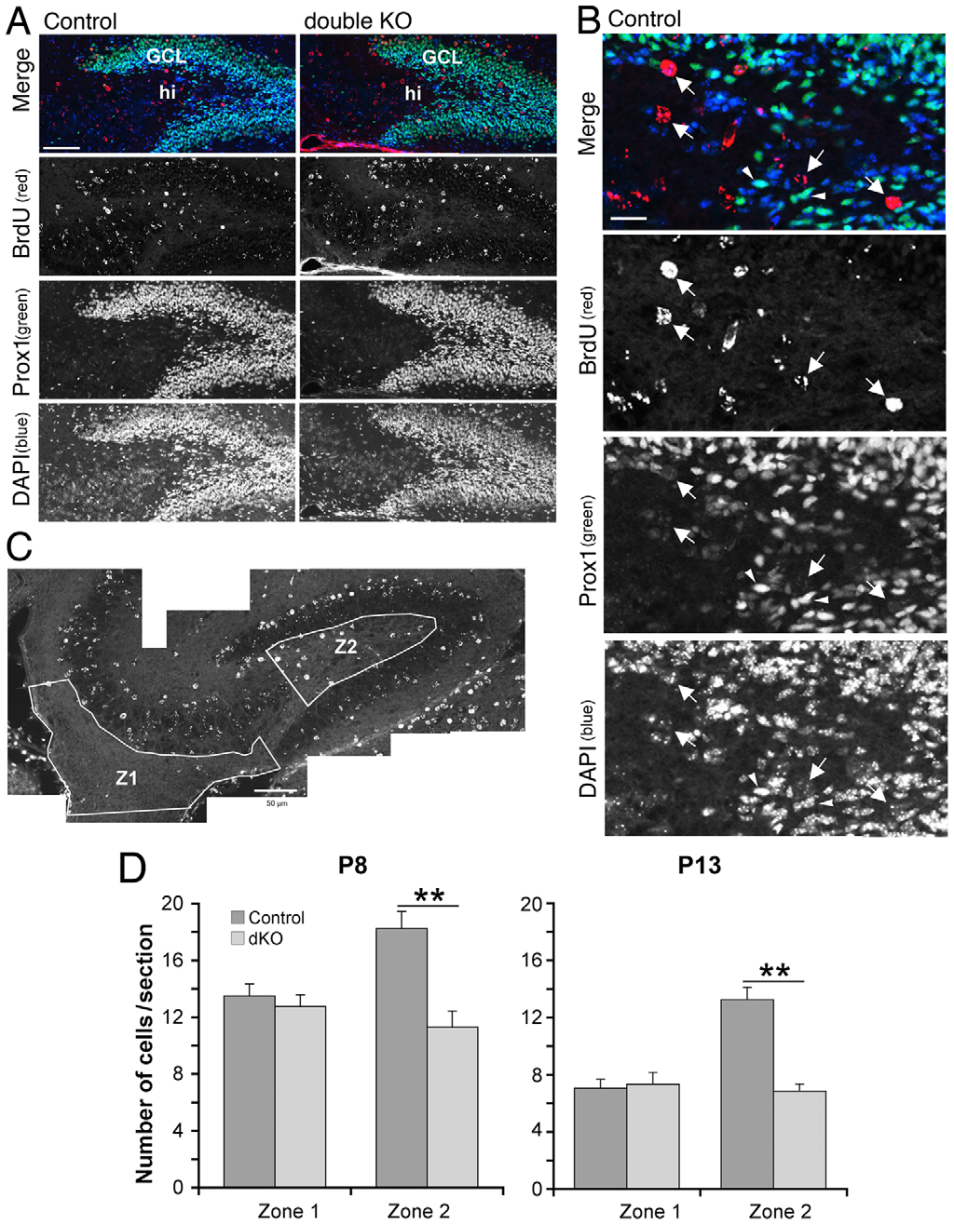
BrdU-positive mossy cell precursors are decreased in the hilus of developing Rac1^N^/Rac3^KO^ mice. Mice were injected twice with BrdU at E12.5–E13 as detailed in the Materials and Methods. BrdU-injected mice were then sacrificed at P8 or P13. (**A**) Sagittal sections of the dorsal hippocampus from P8 Rac3^KO^ (left panels) and Rac1^N^/Rac3^KO^ (right panels) mice were immunostained with antibodies for BrdU (red) and for Prox1 (green); nuclei were visualized by DAPI (blue). GCL, granule cell layer; hi, hilus. Scale bar: 50 µm. (**B**) Enlargement of the hilar region from the control section shown in (**A**). Arrows point to putative mossy cell precursors identified as BrdU-positive, Prox1-negative cells with a large round nucleus. Arrowheads indicate Prox1-positive granule cells. Scale bar: 12.5 µm. (**C**) Hippocampus from a P8 control mouse stained for BrdU. The areas used for quantification are indicated: zone 1 (Z1) includes the ventricular and subventricular zones and the dentate migratory stream; zone 2 (Z2) includes the hilar region, from which the zone that may include pyramidal cells from the CA3 has been excluded. Scale bar: 50 µm. (**D**) Quantification of BrdU-labelled cells in zone 1 and zone 2 of the hippocampus of Rac3^KO^ and Rac1^N^/Rac3^KO^ littermates at P8 and P13. Bars are means ± SEM of the number of BrdU-positive cells (zone 1), and of BrdU-positive, Prox1-negative cells with large round nuclei (zone 2). At each stage, BrdU-positive cells were counted from 24–27 sections taken from 3 different mice per genotype. **P<0.001.

Given the lack of specific markers for developing mossy cell precursors, we decided to look at the effects of the double Rac1/Rac3 knockout on the number of presumptive mossy cell precursors, defined here as the cells located in the hilus that were Prox1-negative, BrdU-positive, and with a large round nucleus (**Fig. 6, A–C**). Prox1 is a marker for dentate granule cells that is not expressed by hilar mossy cells [18], [46]–[48]. We therefore used this marker to distinguish between Prox1-positive granule cells and Prox1-negative mossy cell precursors in the developing hilus. We found that in the hilus, in contrast to the BrdU-positive/Prox1-negative cells that were characterized by a large nucleus, the Prox1-positive granule cells were characterized by a small nucleus (**Fig. 6, B**). We found that the number of putative mossy cells was significantly reduced in the hilus (zone 2) of double knockout mice at both P8 and P13, while the number of total BrdU-positive cells along the precursor migratory pathway (zone 1) was not affected at either age (**Fig. 6, D**). These results indicate that one possible explanation for the decrease of mossy cells in the hilus of the Rac1^N^/Rac3^KO^ mice is a defect in the migration of the precursors to the hilus.

### Conclusions

We have investigated the hilar defect observed in double knockout mice by a comparative analysis between double knockout and control Rac3^KO^ mice. Although the development of hilar mossy cells has been poorly investigated due to the lack of markers for immature mossy cells, recent neuronal birthdating experiments have shown that the majority of mouse mossy cells are born between E12.5 and E13.5 [18].

The precise timing for the migration of mossy cells towards the hilus in not known, and migration may therefore occur any time between their birth (E12.5–E13) and their first identification within the hilar region at the end of the first postnatal week. The absence of known specific early markers for these cells has also prevented the identification of the time of arrival of the immature mossy cells in the hilus. In this study, we have shown that while the number of GluR2/3-positive cells in the dorsal hippocampal hilus increased about two-fold in the control mice between P8 and P13, the number of GluR2/3-positive cells remained about the same at the two ages in the double knockout mice. This finding may be explained by the sustained inhibition of precursor arrival to the hilus that is already detectable at P8. On the other hand a defect in the maturation of the cells that have reached the hilus could also contribute to the strong decrease detected at P13. This is supported by the finding that the number of BrdU-positive, Prox1-negative presumed mossy cell precursors decreased in control mice between P8 and P13 (**Fig. 6D**). Therefore, it seems more likely that the majority of mossy cells has already reached the hilus by P8, while they continue to differentiate during the second postnatal week.

The finding that at P8 only approximately 30% of the BrdU-positive Prox1-negative cells are GluR2/3+ in control mice suggests that mossy cells were still differentiating at P8. On the other hand, differentiation defects could be already observed in the hilus of P8 double mutants, where both presynaptic and dendritic development were affected. This may be at least in part a consequence of the reduction of GluR2/3-positive mossy cells already evident at P8. The correlation between the loss of mossy cells and the loss of axonal inputs and dendritic development in the hilus was confirmed at P13. While mossy cells/precursors did not evidently accumulate in the VZ/SVZ/DMS, BrdU-positive precursors were significantly reduced in the hilus of P8 double knockout mice compared to control littermates (**Fig. 6**). Altogether, these findings suggest that the deletion of both Rac1 and Rac3 GTPases affects the migration of mossy cell precursors to the hilus. The findings that GluR2/3-positive differentiated mossy cells and hilar cells with characteristics of mossy cells were strongly reduced in the hilus of P8 and P13 mice, together with the lack of an evident increase in apoptosis and defect in precursor proliferation support the hypothesis that the lack of mossy cells in the hilus of Rac1^N^/Rac3^KO^ mice is due to a migratory defect of their precursors. Although mislocalized hilar mossy cell precursors may accumulate along the migratory pathway, the relative small number of these cells compared to comigrating granule cell precursors and the lack of known markers for the early development of hilar mossy cells does not allow to directly demonstrate their accumulation in these areas.

Rac3 represents about 10% of the total Rac in the brain, and is expressed only in neurons, while Rac1 is also present in the glia. Given the differential distribution of the two GTPases in different neuronal populations, their relative amounts may vary in different neurons. The findings that hilar mossy cells develop normally in Rac1^N^ and Rac3^KO^ mice [15], and that Rac3 expression is detectable relatively late during neural development [14], indicate that the lack of either Rac may be at least partially compensated by the other GTPase in the Rac1^N^ and Rac3^KO^ mice, respectively. In this direction, pull down experiments from brains of wildtype and Rac3^KO^ mice showed a 24% increase in Rac activation compared to wildtype animals (**Fig. S5**). Similarly, Rac2-deficient B cells showed increased BCR-induced Rac1 activation, suggesting hyperactivation of Rac1 may compensate for the lack of the hematopoietic-specific Rac2 GTPase in this system [49].

Altogether, our results indicate that the specific defect of hilar mossy cells induced by the double deletion of neuronal Rac1 and Rac3 is reflected by a decrease in mossy cell precursors detectable already in early postnatal development, and suggest that the decrease in mature mossy cells is principally due to a defect in the migration of the precursors. Our unpublished data indicate that *SynI-Cre* is not active in the brain until E15.5, while it is active at P0. Therefore, we expect that the combination of the late deletion of Rac1 in Rac3KO neurons may affect mossy cell precursor migration and development. In support of a role of Rac proteins in neuronal migration, a recent study has shown that the deletion of Rac1 in neural progenitors in the ventricular zone caused a defect in the establishment of their migratory competency [12], while the deletion of Rac1 by a *nestin-Cre* caused aberrant migration and defective axon formation in cerebellar granule neurons [50].

Further analysis on the development of this interesting population of hippocampal neurons awaits the identification of specific markers for the immature mossy cells. We believe that these findings may also be relevant to the study of the etiology of epilepsy in neurological and psychiatric diseases linked to aberrant neuronal development.

## Materials and Methods

### Ethics statement

Maintenance and experimental handling of mice were performed after approval of the protocols by the Institutional Committee of the San Raffaele Scientific Institute (approval n. SK 464), according to the Italian law (Decr. Leg. n.116, 27.1.1992, according to the EEC regulation n. 86/609/CEE).

### Mice

The production of Rac3^KO^, and Rac1^N^/Rac3^KO^ mice and genotyping were as previously described [15]. Rac1^N^/Rac3^KO^ double knockout mice and control Rac3^KO^ littermates were used for the experiments described in this study.

### Antibodies and plasmids

The following antibodies and dilutions were used in this study: rabbit pAb anti-GluR2/3, 1∶100 for immunohistochemistry, 1∶50 for immunofluorescence (Upstate); mouse mAb anti-MAP2 (microtubule associated protein 2), 1∶250 (clone HM-2, Sigma-Aldrich); mouse mAb anti-Synaptotagmin 1, 1∶200 (Synaptic Systems); rabbit pAb anti-Prox1 (prospero-related homeobox 1), 1∶1000 (Millipore); mouse mAb anti-BrdU, 1∶100 (BD); rabbit pAb anti-zinc transporter-3 (ZnT-3), 1∶200 (gift of Richard Palmiter) [44]. All fluorescent secondary antibodies (goat anti-rabbit Alexa Fluor 488, goat anti-mouse Alexa Fluor 568, goat anti-mouse IgG1 Alexa Fluor 546, goat anti-mouse IgG2a Alexa Fluor 647) were from Invitrogen and were diluted 1∶200. Biotinylated goat anti-rabbit was from Vector Laboratories and used at 1∶200.

The GST (glutathione-S-transferase)-PAK-CRIB plasmid was a gift from Dr. John Collard [51].

### Morphological analysis

Mice were fixed under deep anesthesia by transcardial perfusion with 4% paraformaldehyde in phosphate-buffered saline. Brains were removed from skulls and postfixed overnight at 4°C. Sagittal frozen 10–14-µm sections were treated for immunofluorescence or immunohistochemistry as previously described [15]. Negative control sections were treated in an identical manner except that the primary antibody was omitted. Sections were analyzed with a Zeiss Axioplan2 microscope equipped with an AxioCam MRc5 digital camera (Carl Zeiss MicroImaging GmbH). Confocal analysis was performed with a Leica TCS SP2 Laser Scanning Confocal microscope (Leica Microsystems GmbH).

### Apoptosis

TUNEL (terminal deoxy-nucleotidyl transferase dUTP nick end labeling) was performed using the ApopTag® Red *In Situ* Apoptosis Detection Kit (Chemicon). Sagittal 12-µm sections were processed following the manufacturer's instructions. Apoptotic indices for the hippocampus of different mouse lines were evaluated as number of apoptotic cells/section of the hippocampal hemispheres. Sections were analyzed with a Zeiss Axioplan2 equipped with an AxioCam MRc5 (Carl Zeiss MicroImaging GmbH). Sections of the interdigit region of E13.5 wildtype mice were used as positive controls [52].

### Cell proliferation and migration

For the analysis of precursor proliferation, pregnant mice at gestational age E12.5 were given a single intraperitoneal injection with BrdU (Sigma-Aldrich) at 100 µg/g of body weight. Females were sacrified 2 hours after injection. Embryos were dissected in ice-cold phosphate-buffered saline, fixed overnight in 4% paraformaldehyde, and processed for immunofluorescence with anti-BrdU antibodies. For cell migration, pulse-chase experiments were performed as follows: pregnant females at gestational age E12.5 were given two intraperitoneal injections with BrdU at 100 µg/g body weight, with a time interval of 6 hours. Double knockout and Rac3^KO^ pups were fixed by perfusion at P8 and P13, and brains were processed for immunostaining for BrdU.

For the detection of BrdU, sections were treated with 2 N HCl for 20 min at 37°C. The pH of the sections was then neutralized by incubation for 10 min in 0.1 M borate buffer, pH 8.5. Sections were equilibrated in 150 mM NaCl, 100 mM Tris-HCl pH 7.5 for 10 min, and then blocked for 1 hour with 3% bovine serum albumin, 1% glycin, 10% goat serum, 0.4% triton X-100 in 150 mM NaCl, 100 mM Tris-HCl pH 7.5. Sections were then processed for immunofluorescence with anti-BrdU antibodies as described in the previous paragraph. Sections were analyzed with a Zeiss Axiovert 135 TV equipped with a QImaging Exi-Blue camera (Carl Zeiss MicroImaging GmbH).

### Quantification and statistical analysis

For quantification of synaptotagmin 1, tissue sections were analyzed with the confocal microscope using a 63× oil-immersion objective. Pinhole was kept constant at one Airy unit, laser power and photomultiplier settings were the same for all samples within the same experiment. Quantification was performed with the ImageJ software (U.S. National Institutes of Health). Synaptotagmin 1 fluorescence was evaluated in the neuropil. The intensity of the signal for synaptotagmin 1 was measured in 8-bit images (1024×1024 pixels) using a grid of 2500 pixels^2^ quadrants. Between 4 to 16 quadrants per section were analysed. The average mean gray value for each section was calculated and individually adjusted for background by subtracting the mean gray value from the immuno-negative nuclei in the same section. Between 2 to 5 sections per mouse were analysed. Values are presented as means ± SEM. Statistical significance (*P*<0.05) was determined by the Student's t test.

### X-Gal (5-Bromo-4-chloro-3-indolyl-ß-D-galactopyranoside) staining

X-gal staining for ß-galactosidase activity was performed on brain slices, as described previously [53] on mice obtained by mating SynI-Cre (Synapsin-I Cre recombinase) mice with LacZ ROSA26 tester reporter mice [54].

### *In situ* hybridization

Postnatal brains were fixed by perfusion and postfixed overnight at 4°C in 4% paraformaldehyde; 20-µm sections were cut after freezing. The probe for Rac3 was prepared and utilized for *in situ* analysis as previously described [6]. The hybridized probe was detected with alkaline phosphatase-coupled antibodies to digoxigenin, according to the manufacturer's protocol (Roche).

### Rac Activation assay

Mouse brains were lysed at 4°C by 5 strokes in a Potter homogenizer in extraction buffer (10% glycerol, 100 mM NaCl, 1% NP40, 2 mM MgCl_2_, 50 mM Tris-Cl, pH 7.4, anti-proteases), and centrifuged to remove the insoluble fraction. Aliquots of brain lysates were incubated for 1 hour at 4°C with 50 µl of glutathione-agarose beads (Sigma-Aldrich) pre-adsorbed with the bacterially purified GST-PAK-CRIB fusion protein including the GTPase-binding domain of the PAK1B kinase [51]. GST-PAK-CRIB-coated beads were washed three times with extraction buffer and analyzed by SDS-PAGE and immunoblotting with anti-Rac antibodies.

## Supporting Information

Figure S1 **Rac3 expression and activation of the** ***SynI-Cre*** **transgene in hilar cells.** (**A**) Expression of Rac3 mRNA in wild-type P13 hippocampus detected by hybridization with a digoxigenin-labelled antisense probe for Rac3. The transcript for Rac3 is expressed by large cells within the hilar region. (**B**) X-Gal staining of a sagittal section of the hippocampus of a P13 SynI-Cre/ROSA26 mouse. The *SynI-Cre* transgene is active in numerous hilar cells. GCL, granule cell layer; hi, hilus. Scale bars: 200 µm.
(TIF)

Figure S2 **The presynaptic input and the dendrites are reduced in the hilus of double knockout mice.** High magnification of the dorsal hilar regions of control (Rac3^KO^) and Rac1^N^/Rac3^KO^ P8 and P13 mice immunostained for the presynaptic marker synaptotagmin 1 (blue), for the dendritic marker MAP2 (red), and for GluR2/3 (green). In control sections mossy cells are surrounded by a dense array of dendrites and axonal terminals that are less dense around mossy cells from double knockout mice. Bars: 20 µm.
(TIF)

Figure S3 **ZnT-3-positive axons are strongly reduced in the hilus of double knockout mice.** Sections from P8 and P13 dorsal hippocampus were stained with antibodies for synaptotagmin 1 (red) and ZnT-3 (green), and with DAPI (blue). Scale bar: 200 µm.
(TIF)

Figure S4 **Cell death in postnatal hippocampus.** Sagittal brain sections from different postnatal stages were used for TUNEL staining (red). Nuclei are shown by DAPI staining (blue). Scale bar: 200 µm.
(TIF)

Figure S5 **Deletion of Rac3 leads to increased Rac1 activation in mouse brain.** (**A**) Active GTP-bound Rac proteins were recovered from lysates of wildtype P7 brain, by pull-down on beads coupled to GST-PAK-CRIB. Lane 1, 86 µg of unbound fraction (Ub) after pull down from wildtype brain lysate (WT); lane 2, 93 µg of the unbound fraction after pulldown from Rac3^KO^ P7 brain lysate (KO); lane 3, positive control: pulldown from 100 µg of wildtype P7 brain lysate loaded *in vitro* with GTPγS; lane 4 and 5, pull downs from 3 mg of brain lysates from P7 brain of wildtype and Rac3^KO^ mice, respectively. Filters blotted for Rac1 (upper), and Rac3 (lower). (**B**) Quantification from two independent experiments of Rac-GTP from wildtype brains (including both Rac1-GTP and Rac3-GTP), and from Rac3^KO^ brains (including only Rac1-GTP).
(TIF)
