# Supplementary figures and images for: Rac1 and Rac3 GTPases Regulate the Development of Hilar Mossy Cells by Affecting the Migration of Their Precursors to the Hilus
Source: PLoS One. 2011 Sep 20;6(9):e24819. doi: 10.1371/journal.pone.0024819 (PMC3176786; doi:10.1371/journal.pone.0024819)

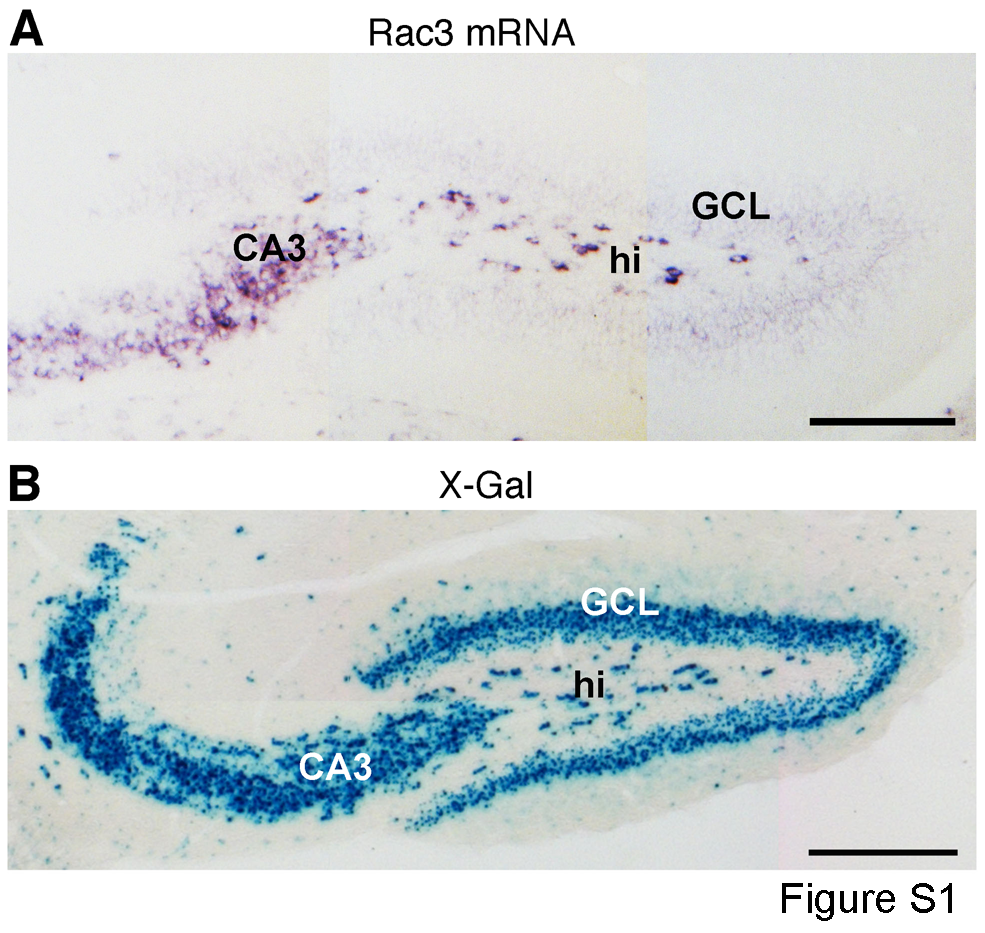

Supplement: Figure S1 — Rac3 expression and activation of the SynI-Cre transgene in hilar cells. (A) Expression of Rac3 mRNA in wild-type P13 hippocampus detected by hybridization with a digoxigenin-labelled antisense probe for Rac3. The transcript for Rac3 is expressed by large cells within the hilar region. (B) X-Gal staining of a sagittal section of the hippocampus of a P13 SynI-Cre/ROSA26 mouse. The SynI-Cre transgene is active in numerous hilar cells. GCL, granule cell layer; hi, hilus. Scale bars: 200 µm. (TIF) [file pone.0024819.s001.tif]

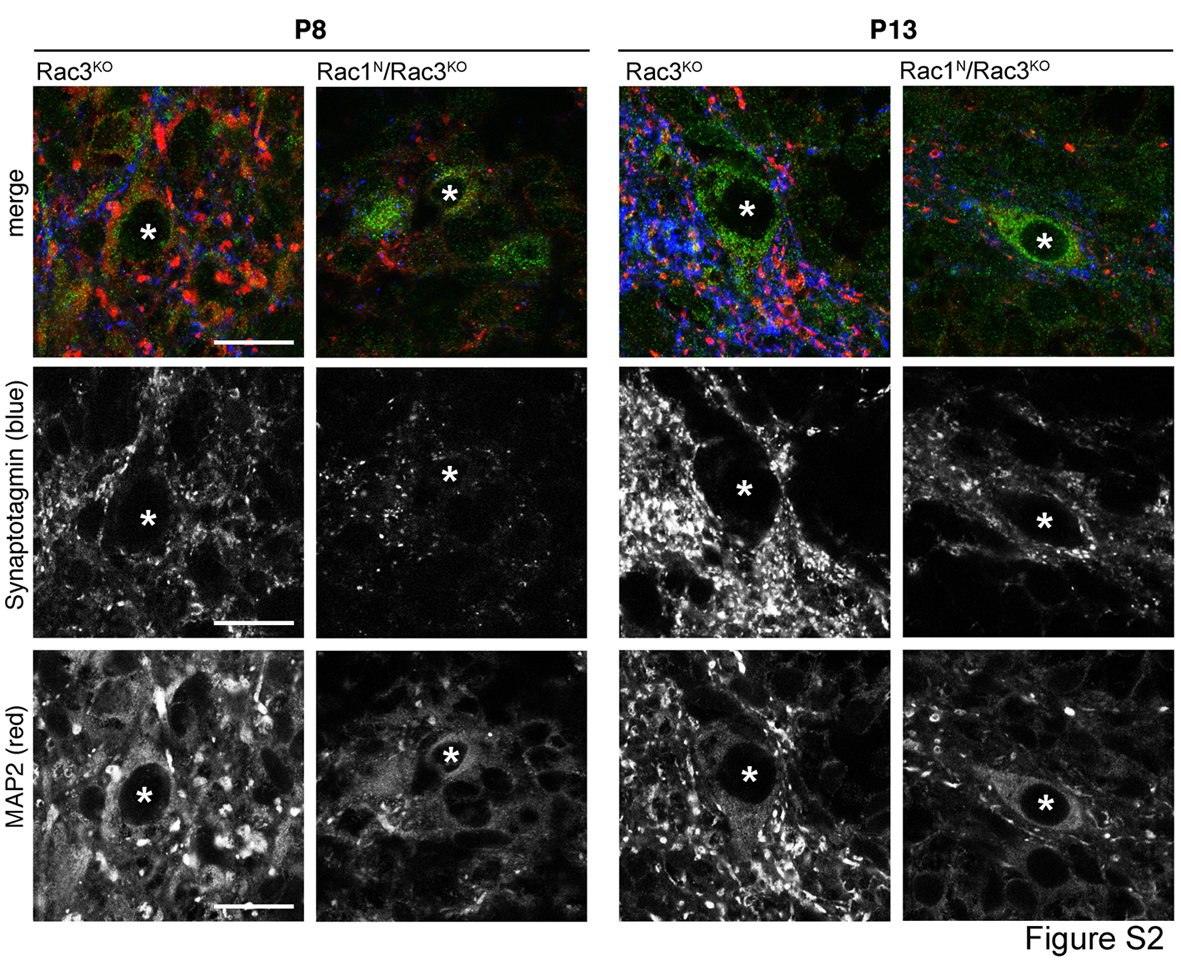

Supplement: Figure S2 — The presynaptic input and the dendrites are reduced in the hilus of double knockout mice. High magnification of the dorsal hilar regions of control (Rac3KO) and Rac1N/Rac3KO P8 and P13 mice immunostained for the presynaptic marker synaptotagmin 1 (blue), for the dendritic marker MAP2 (red), and for GluR2/3 (green). In control sections mossy cells are surrounded by a dense array of dendrites and axonal terminals that are less dense around mossy cells from double knockout mice. Bars: 20 µm. (TIF) [file pone.0024819.s002.tif]

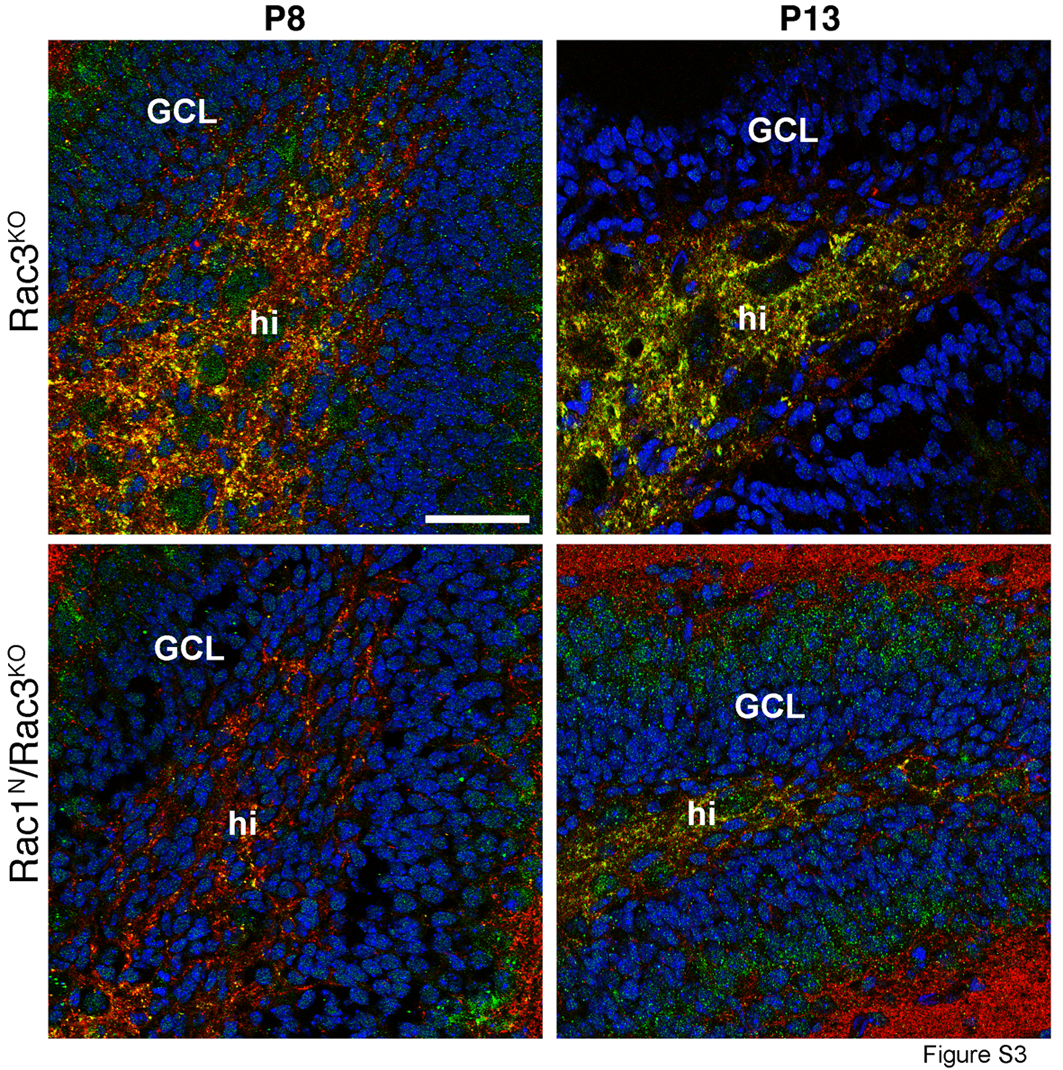

Supplement: Figure S3 — ZnT-3-positive axons are strongly reduced in the hilus of double knockout mice. Sections from P8 and P13 dorsal hippocampus were stained with antibodies for synaptotagmin 1 (red) and ZnT-3 (green), and with DAPI (blue). Scale bar: 200 µm. (TIF) [file pone.0024819.s003.tif]

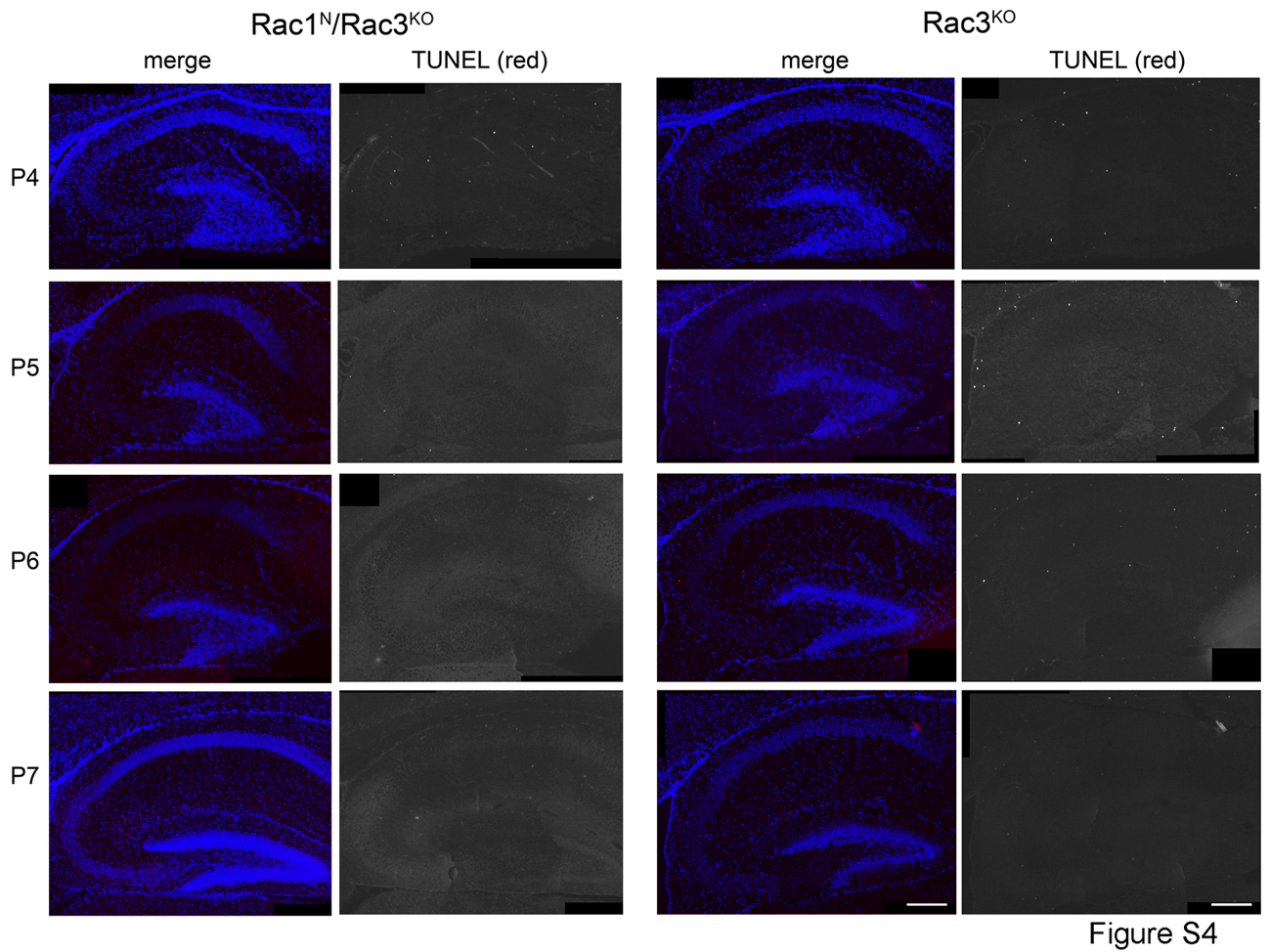

Supplement: Figure S4 — Cell death in postnatal hippocampus. Sagittal brain sections from different postnatal stages were used for TUNEL staining (red). Nuclei are shown by DAPI staining (blue). Scale bar: 200 µm. (TIF) [file pone.0024819.s004.tif]

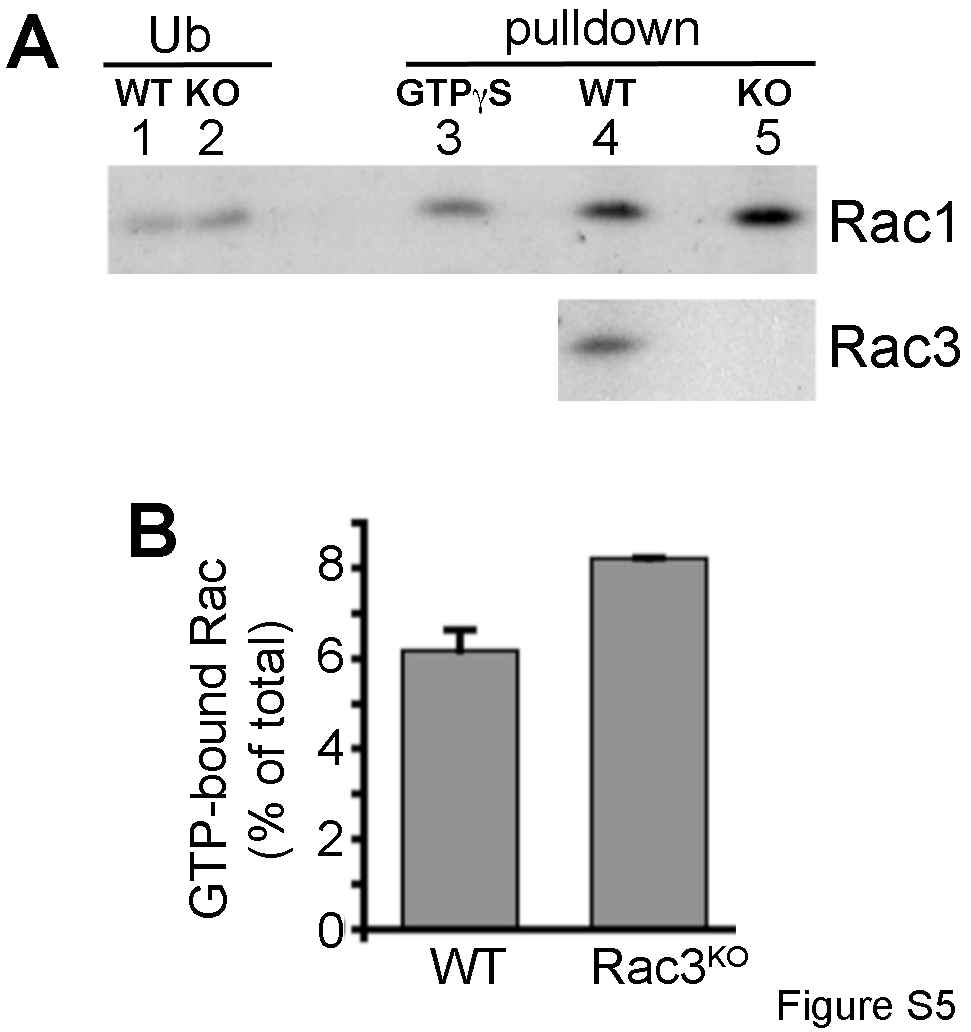

Supplement: Figure S5 — Deletion of Rac3 leads to increased Rac1 activation in mouse brain. (A) Active GTP-bound Rac proteins were recovered from lysates of wildtype P7 brain, by pull-down on beads coupled to GST-PAK-CRIB. Lane 1, 86 µg of unbound fraction (Ub) after pull down from wildtype brain lysate (WT); lane 2, 93 µg of the unbound fraction after pulldown from Rac3KO P7 brain lysate (KO); lane 3, positive control: pulldown from 100 µg of wildtype P7 brain lysate loaded in vitro with GTPγS; lane 4 and 5, pull downs from 3 mg of brain lysates from P7 brain of wildtype and Rac3KO mice, respectively. Filters blotted for Rac1 (upper), and Rac3 (lower). (B) Quantification from two independent experiments of Rac-GTP from wildtype brains (including both Rac1-GTP and Rac3-GTP), and from Rac3KO brains (including only Rac1-GTP). (TIF) [file pone.0024819.s005.tif]
